# Wake up! Messrs. Walter Long and Winston Churchill

**Published:** 1919-02-01

**Authors:** 


### GRAVE INJUSTICES TO MEDICAL MEN.

### Wake Up! Messrs. Walter Long and Winston Churchill.

Demobilisation of Medical men is not proceeding in a way satisfactory to those most intimately concerned. No class of the community has made heavier sacrifices to the common cause than the doctors. The sacrifice was made willingly because the nation was in need. Unfortunately, the medical profession is not a corporation, and has no union of its parts, and therefore is at the mercy of the remainder of the community. It has not the means to speak with a collective voice, nor the habit of doing so. Therefore the interests of its members were never considered, and are not being greatly considered now.

Men in good practice threw up all to take a very meagre pay in comparison with their earnings in civil life. Whilst miners and munition workers were able to earn £500 a year or so, and to live in comfort with their families, hundreds of medical men were drawing less than these, and had to keep on expensive houses necessitated by their practices, to pay the expenses of their wives and families, and to see the practices in which they had sunk their capital to buy, and which they had built up by many years of hard and anxious work, rapidly disappear as their old patients left the locality and the newcomers perforce chose the man who remained behind. Very many men have had to draw on savings to pay the difference between their receipts from a country grateful perhaps, but still not disposed to show itself grateful to servers of it, who could not or would not make themselves unpleasant by concerted action. "Threaten us and we will raise your pay; do your best and keep silent, and we will forget you" has been the attitude of the nation. That is why the junior infantry officer gets less than many a girl in a country aircraft factory.

And now everything is being done to facilitate the demobilisation of every organised class of the community, and large pecuniary inducements are being offered to every class of man to stay in the Army, but the doctor is told he has got to stay, whether he is forty years old or no, and whether he likes it or no, and to get nothing extra for staying. Further, the regular officer is told that though the sovereign is, and has been only worth twelve shillings, he cannot have any bonus for the war period, though every other wage-earner has had it, and extra wages to boot. If medical men had a good strong Trade Union they would be as well able as policemen, engineers, miners, and dock-hands to get consideration of their demands, demands that would be at least as reasonable as those of other classes of the community.

Meanwhile, it is certain that the people at large, when they become aware of the shabby treatment at present extended to members of the medical profession, who sacrificed their interests and have done splendid work in the saving of life, and the treatment and restoration to health of our warriors throughout this war, will certainly not hold blameless the members of the Ministry as a whole, and especially the First Lord of the Admiralty and the Secretary of State for War, unless they take immediate steps to inquire into the whole position. It is a primary public duty to secure generous treatment and the fullest consideration for the members of the medical services, who have so fully met the requirements of the nation, and whose work has had such momentous results not only in saving life, but in reducing the cost of the war and contributing materially to its shortening. The Government must sympathise with and sustain the medical profession, by making it possible for the best of its members to dispense with trade union methods, which are distasteful to educated men of intellect and prowess. Are we to be compelled to believe that the deadening effect of high office at the Admiralty and War Office is so all-embracing as to exclude the exercise of their highest faculties by the men in whose hands it really rests to secure generous and adequate treatment for British doctors and surgeons of all grades? The present position is a lamentable exhibition of the impotence of the British Medical Association and those who at present control it. They must be held responsible for many of the evils it has been our duty to here set forth, including neglect of the paramount claims of our physicians and surgeons.

